# Application of Molecular Imprinting for Creation of Highly Selective Sorbents for Extraction and Separation of Rare-Earth Elements

**DOI:** 10.3390/polym15040846

**Published:** 2023-02-08

**Authors:** Ruslan Kondaurov, Yevgeniy Melnikov, Laura Agibayeva

**Affiliations:** 1 Department of Biochemical Engineering, International Engineering and Technological University, Al-Farabi ave. 93a, Almaty 050060, Kazakhstan; 2 Faculty of Chemistry and Chemical Technology, Al-Farabi Kazakh National University, Al-Farabi ave. 71, Almaty 050040, Kazakhstan

**Keywords:** light and heavy rare-earth metals, selective sorption, separation of rare-earth metals, molecular imprinting technique, functional macromolecular structures, molecularly imprinted polymers

## Abstract

The aim of the work is to study the effectiveness of a molecular imprinting technique application for the creation of highly selective macromolecular sorbents for selective sorption of light and heavy rare-earth metals (for example, samarium and gadolinium, respectively) with subsequent separation from each other. These sorbents seem to be promising due to the fact that only the target rare-earth metal will be sorbed owing to the fact that complementary cavities are formed during the synthesis of molecularly imprinted polymers. In other words, the advantage of the proposed macromolecules is the absence of accompanying sorption of metals with close chemical properties. Two types of molecularly imprinted polymers (MIP) were synthetized based on methacrylic acid (MAA) and 4-vinylpyridine (4VP) functional monomers. The sorption properties (extraction degree, exchange capacity) of the MIPs were studied. The impact of template removal cycle count (from 20 to 35) on the sorption effectivity was studied. Laboratory experiments on selective sorption and separation of samarium and gadolinium from a model solution were carried out.

## 1. Introduction

The market for rare-earth metals (REM) is one of the youngest commodity markets in the world and is growing at an impressive pace compared to other base metals (nickel, copper, iron, gold, etc.): over the last 50 years, the volume of world production and consumption of REMs increased by about 40 times—from 5000 to 200,000 tons per year [1,2]. This was the result of both global economic growth and a change in technological structures based on the innovative development of the world economy. The volumes of production and consumption of REMs are one of the main signs of the development of the national industry of a country and a significant indicator of its manufacturing ability and innovative component. It is rare-earth metals that in the last decade have caused the greatest concern among developed and developing countries due to their strong integration into the production chains of high-tech industries and the level of uncertainty involved in providing this type of raw material [3]. At the same time, in addition to the global geopolitical situation, the rare-earth industry is developing naturally due to scientific and technological progress and free competition [4]. There are new technologies and innovative products (and hence new demand), to which companies respond by modifying and reducing the cost of the production processes of their products. In this regard, an important aspect of the development of a national rare-earth industry is such institutional conditions for suppliers of rare-earth raw materials, its consumers and a state that would protect “their” enterprises and be able to address various crises and the destruction of existing global production chains [5,6,7].

It is necessary to highlight the following features that determine the relevance and demand for REMs at the present time:

1. REMs in the Earth’s crust are not relatively rare; they are more common than, for example, gold, uranium, lead, tin, molybdenum, tungsten, etc. However, deposits with industrial concentrations of rare-earth ores are less common than for most other minerals. According to the report “Strengthening the European rare earths supply-chain”, the available REM reserves exceed the current world production by three orders of magnitude [8].
2. Ores are complex in composition. In addition to REMs, they contain elements such as niobium, tantalum, phosphorus, iron, aluminum and others. More than 250 minerals are known that contain REMs, but only 60–65 of them are rare-earth. All rare-earth deposits differ greatly in their specific distribution of metals. As a rule, light rare-earth metals (LREM) make up a much larger proportion of the total content of REMs in the ore than heavy rare-earth metals (HREM). Therefore, in our time, one of the most important tasks is still the development of fundamentally new approaches and technologies for deep and complex processing of complex rare-earth ores that cannot be enriched by traditional physical and mechanical methods. It is for this reason that recently, more and more often in the media, one can find reports on research in the field of search and extraction of REMs from such potential sources as various industrial wastes, tailings and slags (for example, ash and slag dumps, phosphogypsum, red mud). A high value is given to deep-sea rocks and silts from the bottom of the southeastern and central parts of the Pacific Ocean, which, according to various estimates, may contain amounts comparable to or even exceeding continental reserves of REMs [9,10].
3. The third feature is that rare-earth ores contain radioactive thorium and uranium, the concentrations of which are very different for each rare-earth deposit. These elements are considered to be byproducts of mining, and the presence of thorium and uranium in the ore is one of the key factors affecting the attractiveness of a deposit to investors, as these two elements can be the biggest barrier to obtaining permission to mine and process the ore. In this regard, special attention is paid to such issues as radioactive dust and radiation in deposits, radioactive waste management and transportation of rare-earth ore, which must comply with strict regional and international legislative standards [11,12].
4. The fourth feature of the REM sources is that rare-earth elements are often byproducts of mining and processing of ore with elements such as iron, cobalt, manganese, titanium, niobium, tantalum, zirconium and others [13,14]. However, the technologies for capturing and separating associated components are complex and unique for each source of mineral resources; therefore, they have no analogues and are expensive. For this reason, small industrial concentrations of REMs relative to other elements in a deposit may turn out to be untenable during a feasibility study, which will not allow the development and operation of a REM source to begin [14].
5. The fifth feature of REMs that needs to be distinguished is the balance problem, or the balancing problem. As in the case of uranium and thorium, the concentrations of which are very different in the ore object, the specific distributions of metals also differ significantly for each REM deposit [15,16,17,18]. Moreover, this distribution does not correspond to the demand of the global market for various types of high-tech products, the production of which requires REMs. The essence of the problem lies in the fact that the mined ore at a deposit is completely processed at the first stages of enrichment into a concentrate without residues and non-selectively. Such a “natural binding” of REMs leads to an excess supply of some of the rare-earth elements and, accordingly, a decrease in prices for them. On the other hand, there is an increased demand for scarce REMs from the market of high-tech products, for the production of which these REM are needed, which leads to an increase in their prices; therefore, surplus REMs are implicitly subsidized by demand at the expense of scarce ones [19].
6. The sixth and last feature of REMs is the change in the dominant area of consumption due to scientific and technological progress, which dramatically changes the demand for REMs and unbalances the market [20,21,22,23,24,25,26]. A change in the dominant area of consumption brings the market and industry out of balance, and the demand for individual REMs changes dramatically, which leads to significant changes in prices and supply chains and an increase in uncertainties and risks, including for investors. Therefore, it is important to understand the dynamics of world demand and the structure and distribution of technological chains for the production of high-tech products based on REMs, which will undoubtedly undergo changes in the foreseeable future [27,28].

**Figure 1:**
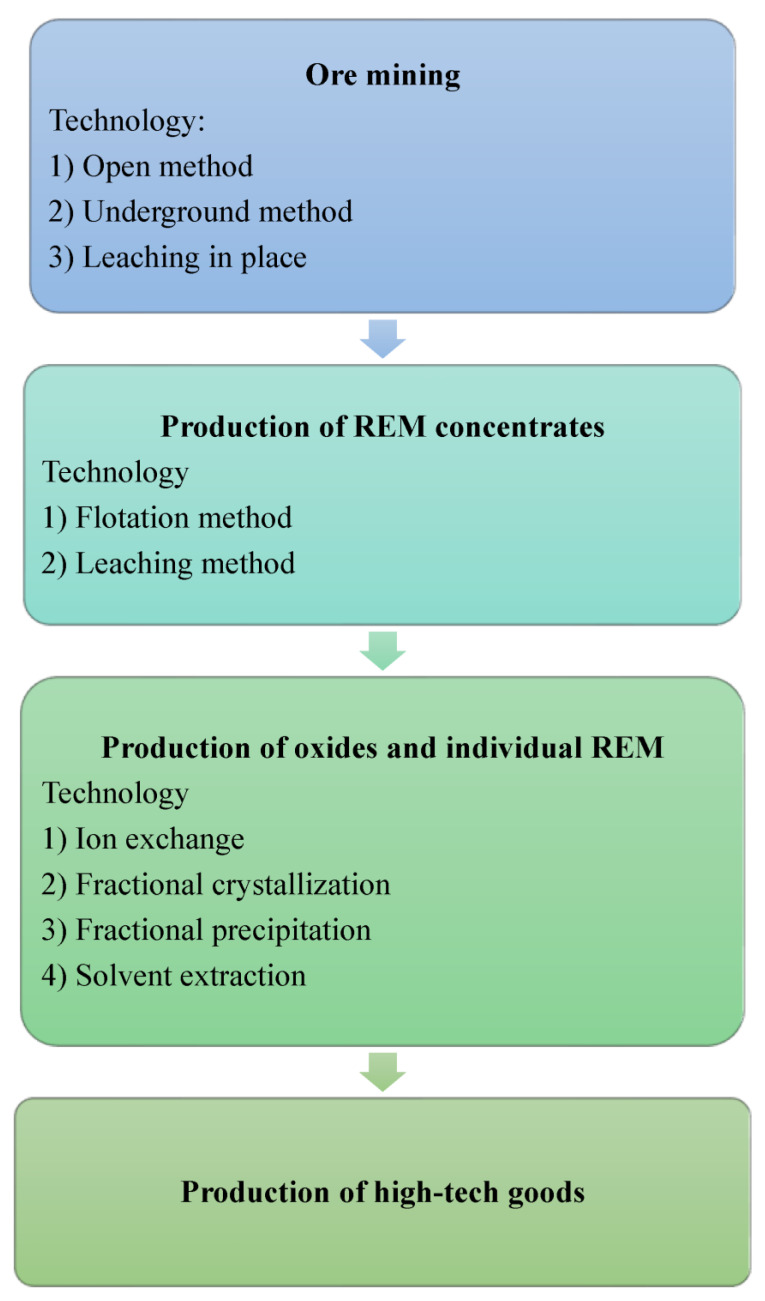
The structure of the technological chain for the production of high-tech products based on REMs.

Figure 1 shows the structure of the technological chain for the production of high-tech products based on REMs [29]. In general, there are three main methods of extracting ore from a deposit: open-pit shallow ore body mining, underground mining and in situ leaching. After the excavation of the rock, due to the complexity of the composition of rare-earth ores, at the second stage, individual (sometimes unique) physical and chemical processing schemes are used, as a result of which various concentrates and intermediate products are obtained at the output. In particular, the initial and intermediate rare-earth products are various concentrates: fluorides, chlorides and carbonates. At the third stage of the production chain, REM oxides are obtained after chemical treatment, from which individual metals are extracted after extraction. Due to the chemical similarity of rare-earth metals, separation into individual metals is a laborious task. Currently, ion exchange and solvent extraction are the two advanced methods for separating concentrates.

Because rare-earth deposits are multicomponent, the technological chain of production “from ore mining to obtaining individual metals” is a multi-stage process. At the same time, each deposit is unique in terms of the composition and content of REEs in the ore, which means that the multi-stage production chain has certain individual technological features for each type of ore. At the same time, there are stages at which similar finished products are obtained: concentrates of different levels (for example, fluorides, chlorides and carbonates of rare-earth metals), oxides or individual metals.

The production chain does not end there, as high-tech goods can be obtained based on REMs and their oxides. It is the possibility of producing such high-tech goods based on rare-earth mineral resources that is a litmus test of the level of a technological structure and the development of a country’s industry [1,2,19].

As an alternative to the available sorption technologies based on the use of ion-exchange resins [30], it is possible to use molecular imprinting for the selective sorption and subsequent separation of target REMs. As is known, molecular imprinting is a method for obtaining “molecular imprints” based on the polymerization of functional monomers in the presence of specially introduced target template molecules [31,32,33]. It is known that molecular recognition is based on the spatial correspondence of structures and non-covalent interactions, namely, electrostatic, hydrophobic, van der Waals, π-π- and cation-π-interactions, as well as hydrogen bonds [34]. At the same time, the combination of the selectivity of specific complexation with strength and the ability to rapidly reverse changes is characteristic of almost all macromolecular structures [35]. Molecular recognition is one of the basic concepts of supramolecular chemistry, which differs from the usual binding between molecules by high selectivity. Molecular recognition is based on the presence on one molecule (the receptor, or “host”) of a site of selective binding to another molecule (the ligand, or “guest”). To do this, the receptor and the ligand must show complementarity, that is, structurally and energetically correspond to each other. The concept of complementarity includes the correspondence of the imprint to the template both in size and shape, and in the presence of complementary functional groups in the imprint that are capable of interacting with the functional groups of the template molecule [36,37,38]. Thus, the ability of molecularly imprinted polymers to recognize a target is based on the conformity of the shape of imprints and the specific functional groups within them to template molecules [39].

The list of modern macroporous sorbents based on synthetic polymers is quite extensive, and most of them are polymethacrylate matrices. The obvious advantages of monolithic sorbents based on synthetic polymers are the relative ease of synthesis and the possibility of varying functional monomers depending on the objectives of the study [40,41].

The essence of the method for obtaining molecularly imprinted polymers (MIPs) is the formation of a stable prepolymerization complex between a template molecule and a functional monomer (in other words, preorganization occurs) by mixing them in a suitable solvent [42,43,44,45,46]. Next, polymerization is carried out in the presence of a cross-linking agent, during which the pre-polymerization complex is rigidly fixed in the polymer network. At the end of the process, template molecules are removed from the cross-linked polymer matrix. As a result, “imprints” (the so-called “imprint sites”) are formed in the polymer, which are cavities that are complementary to the template molecule in size, shape and arrangement of functional groups [47]. Briefly, the process of pore formation in MIPs can be described as follows. After the decomposition of the initiator at the initial stage of polymerization, gel-like oligomeric particles (cores) are formed, which begin to precipitate from the organic phase due to low solubility in porogens. Under such conditions, the monomeric part of the organic phase is the best solvent for nascent polymer chains compared to the porogen phase, which facilitates the penetration of monomers into the precipitated insoluble nuclei and their continued participation in the polymerization process occurring inside the nuclei, which gradually reach the size of microglobules. Growing polymer globules are combined into clusters held by polymer chains penetrating neighboring particles. At the final stage of polymerization, the size of the clusters becomes sufficient for their contact, which leads to the formation of a continuous array inside the polymerizing system. The resulting matrix is gradually strengthened by interglobular cross-links and ongoing polymerization. In this case, the formation of a final porous polymeric material is achieved. At this stage, porogenic solvents are a separate organic phase that fills the voids of the porous polymer mass. The fraction of voids (or macropores) in the final polymer is close to the volume fraction of thoracic solvents in the initial polymerization mixture [48,49,50,51,52,53].

**Figure 2:**
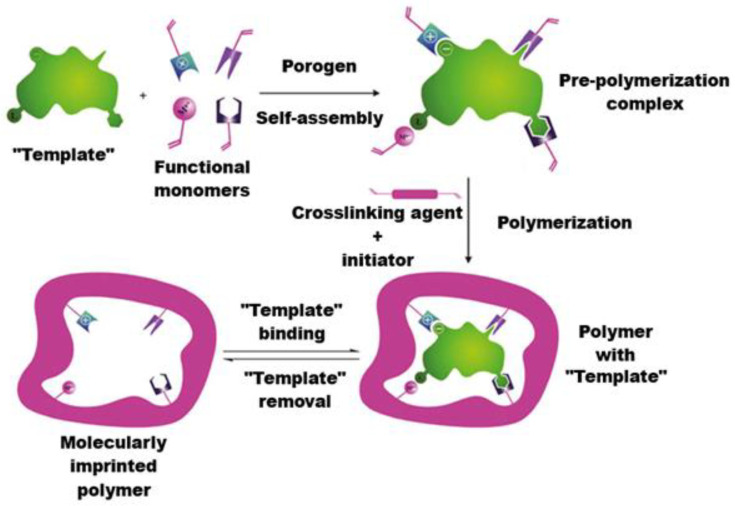
MIP synthesis stages.

Thus, an MIP is essentially a solid matrix with artificial receptors of the template molecule capable of repeated highly specific interaction with it or with its analogue [54]. Schematically, the process of obtaining an MIP is shown in Figure 2 [55].

In this paper, we propose a variant for the development of polymers with molecular imprints as an alternative to existing sorbents for the purpose of their further application for the selective extraction and separation of rare-earth metals. From the light and heavy REMs, samarium and gadolinium were selected due to their relevance to many areas of modern life. Samarium is widely used in the following spheres: magnetic materials; thermoelectric materials; and production of special luminescent and infrared-absorbing glasses [56,57,58,59,60,61]. Gadolinium is mainly used in the following areas: creation of storage media with enormous recording density; nuclear energetics; thermoelectric materials; and superconductors [62,63,64,65].

## 2. Materials and Methods

### 2.1. Materials

The following reactants were used for the synthesis of molecularly imprinted polymers: monomers—methacrylic acid (MAA) and 4-vinylpyridine (4VP); cross-linkers—ethyleneglycol dimethacrylate (EGDMA) and diethyleneglycol dimethacrylate (DEGDMA); initiator—azobisisobutyronitrile (AIBN); porogen—toluene; and stabilizer—hydroxyethylcellulose (HEC). The mentioned reagents were purchased from Sigma-Aldrich (Burlington, MA, USA).

All conducted experiments involved the application of deionized water (χ = 12 µS/cm; pH = 6.95).

Before the experiments the MAA and 4VP monomers were initially purified from inhibitors (monomethyl ether of hydroquinone and hydroquinone) by vacuum distillation.

### 2.2. Methods

#### 2.2.1. Synthesis of Molecularly Imprinted Polymers

**Figure 3:**
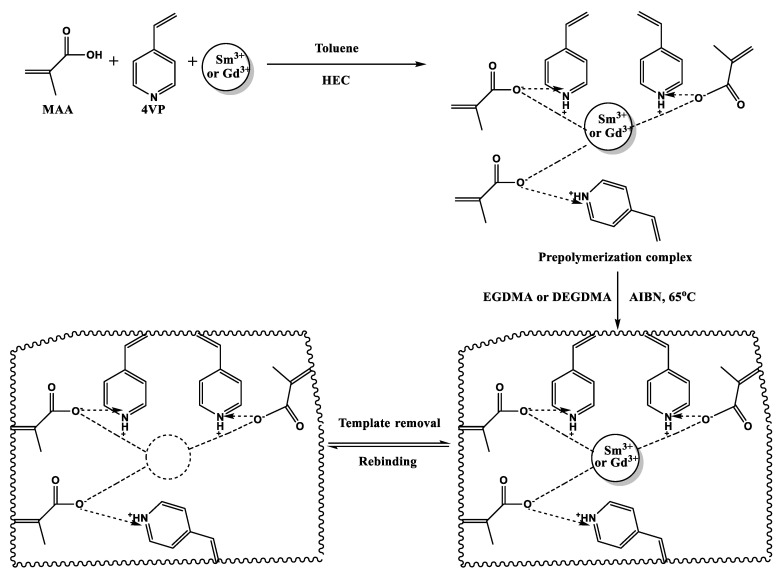
Scheme of the MIP synthesis.

Suspension polymerization was used for the synthesis of molecularly imprinted polymers (MIP). The template molecules were hexahydrate nitrates of gadolinium and samarium. The reactive medium was deionized water. The monomers MAA and 4VP were put into the reactor (containing deionized water) after a template molecule was added. Subsequently, the pre-polymerization complex was added with AIBN, EGDMA (or DEGDMA), tolyene and HEC (polymerizate composition was as follows: template:MAA:4VP:cross-linker = 0.5:1:1:4). Initially the polymerization process occurred at room temperature (for 20 min), but further reaction was carried out at 75 °C in a nitrogen atmosphere with permanent stirring. The obtained imprinted structures are named MIP1 and MIP2, depending on the cross-linker; EGDMA was used in the synthesis of MIP1, and DEGDMA was used in the synthesis of MIP2. The obtained MIPs were crushed into small dispersions and divided by sieving (for further experiments, the particles 200 ≤ d ≤ 250 µm were taken). After that, the particles were washed firstly with deionized water and after that with acetone to remove impurities and unreacted monomer residues. The subsequent procedure was vacuum drying (for 48 h). Removal of the template from the polymer matrix of the MIP was accomplished by continuous washing with nitric acid (concentration 1 mol/L) for 40 repetitions (each cycle—washing with stirring for 1 h), with further washing and drying for 24 h. For proof that the synthesized MIPs have selectivity for the REMs, non-imprinted structures were synthetized along with the MIPs. The synthesis procedure for the non-MIPs is similar to that mentioned above, except the template molecule is not added to the polymerizate. The scheme of synthesis of the MIPs is presented in Figure 3.

Control of purification of the obtained MIPs and full template removal from the MIP matrix was achieved by using an Expert-002-2-6-p conductometer (Econics, Mocsow, Russia) anda SevenDirect SD50 pH meter (Mettler-Toledo, Columbus, OH, USA) for measurements of specific electric conductivity and pH values. The procedure continued until constant values of specific electric conductivity and pH were reached.

#### 2.2.2. Sorption Experiments

Initially, solutions of REM salts were prepared—hexahydrate nitrates of gadolinium and samarium with concentrations of 100 mg/L. The previously mentioned MIP dispersion (0.12 g) was put into the solutions (200 mL) for 2 days (48 h). The temperature in the laboratory during the sorption experiments was 25 °C. Aliquots of the solutions were sampled at certain intervals, which was necessary for further determination of residual concentrations of REMs.

#### 2.2.3. Study of the Synthetized MIP Selectivity

Laboratory experiments devoted to selective sorption of REMs with their further separation were carried out using a developed installation based on two blocks. Each of the blocks contains cartridges for macromolecular dispersion of MIPs. The first block is filled with a dispersion of MIP-Sm, while the second one is filled with MIP-Gd. The studies were carried out as follows: the model solution (contains Sm and Gd, concentration of each REM is 100 mg/L) is pumped into the 1st block for 48 h (sorption of Sm). Subsequently the solution is pumped into the 2nd block for 48 h (sorption of Gd). During sorption of Sm and Gd, aliquots are sampled at certain intervals. After the sorption/separation process ends, the cartridges can be removed and exchanged with other cartridges containing MIP structures, and the installation is ready to begin a new sorption/separation cycle.

For pumping the model solution, a KNF N 816-3 KT-18 laboratory membrane vacuum pump (KNF, Hamburg, Germany) was used.

#### 2.2.4. Study of Impact of Template Removal Duration on the Sorption Capacity Regeneration Process

The study of the influence of the template removal duration on the efficiency of the sorption capacity regeneration process was carried out as follows:

After sorption of the REMs (Sm and Gd) for 48 h, the structures MIP-Sm and MIP-Gd underwent template removal (desorption) as was described above for the following different times: 20, 25, 30 and 35 h. Subsequently these structures were used for another sorption cycle, and solution aliquots were sampled.

#### 2.2.5. Measurement of Residual Concentrations of Gd and Sm

The residual concentrations of the REMs was determined by the photocolorimetric method and atomic emission spectroscopy; a KFK-3-01 photocolorimeter (ZOMZ, Sergiyev Posad, Russia) and Optima 8300 ICP-OES spectrometer (Perkin-Elmer, Santa Clara, CA, USA) were used.

### 2.3. Sorption Parameters Calculation

The following sorption parameters are calculated based on the residual concentrations of Sm and Gd in the solution:

(1) Sorption (extraction) degree [66]:

$$\eta =\frac{C_{0}-C_{e}}{C_{0}}*100\%$$

where *C*_0_ is the initial concentration of the REM (mg/L); and *C_e_* is the initial (equilibrium) concentration of the REM (mg/L).

(2) Exchange capacity [66]:

$$Q=\frac{m_{sorbed}}{m_{sorbent}}$$

where *m_sorbed_* is the mass of the sorbed REM (g); and *m_sorbent_* is the mass of the MIP (g).

(3) REM medium sorption efficiency after sorption/desorption cycle:

$$SE_{pi}=\frac{\sum {\left(\frac{P_{i}}{P_{0}}\right)}_{m}}{n}*100\%$$

where *p* is the sorption parameter (sorption degree or exchange capacity); *i* is the cycles of template removal; m is the time of aliquot taking; *n* is the number of times aliquots were taken; *P_i_* is the MIP sorption parameter after *i* cycles of template removal; and *P*_0_ is the initial MIP sorption parameter.

(4) Growth of the sorption parameters depending on amount of template removal cycles:

$$\omega_{p}=100\%-SE_{pi}$$

where *p* is the sorption parameter (sorption degree or exchange capacity); *P_i_* is the MIP sorption parameter (sorption degree or exchange capacity) after *i* cycles of template removal; and *P*_0_ is the initial MIP sorption parameter.

(5) Sorption parameter medium growth:

$$\varpi =\frac{\sum P_{i}}{i}=\frac{\omega_{\eta }+\omega_{Q}}{2}$$

where *ω_η_* is the sorption degree growth (%); and *ω_Q_* is the sorption capacity growth (%).

## 3. Results and Discussion

The interaction of the synthesized MIPs with samarium and gadolinium salts leads to the sorption of these metals. The sorption character changes with time, and there is the appearance of areas of intense sorption which changes to a slight increase when approaching the equilibrium state between the macromolecular structure and salt solution.

### 3.1. Sorption of Sm and Gd

Synthetized imprinted structures MIP1-Sm, MIP1-Gd, MIP2-Sm and MIP2-Gd interact with nitrates of samarium and gadolinium, resulting in the sorption of these metals.

**Figure 4:**
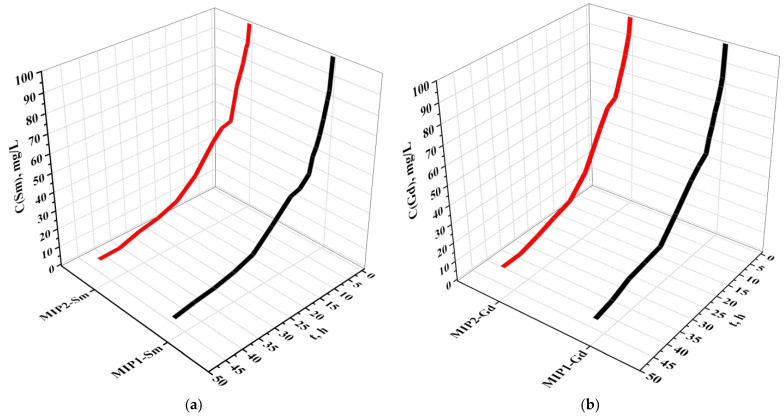
Decrease of samarium (**a**) and gadolinium (**b**) concentrations during sorption via molecularly imprinted polymers.

Figure 4 presents decreases of the the samarium (a) and gadolinium (b) concentrations during sorption by the imprinted structures. Concentrations of the REMs decrease with time of interaction of the MIPs with the corresponding nitrates. In the case of Sm sorption, the concentration of the metal decreases from 100 mg/L–38.99 mg/L (for MIP1-Sm); for MIP2-Sm, the concentration of Sm decreases from 100 mg/L–46.45 mg/L during first 12 h of interaction. The mean difference of the Sm concentration decrease in this time interval (from 0 h to 12 h) for the structures MIP1-Sm and MIP2-Sm is 9.42 mg/L. The further decrease of the Sm concentration is not so intense for the both the imprinted structures; the mean difference of the Sm concentration decrease in this time interval (from 12 h to 48 h) for the structures MIP1-Sm and MIP2-Sm is 4.06 mg/L. In the case of Gd sorption, a strong decrease of the metal concentration is observed during 12 h after the beginning of the contact. The Gd concentration decreases from 100 mg/L to 42.33 mg/L for MIP1-Gd and from 100 mg/L to 52.57 mg/L for MIP2-Gd. The mean difference of the Gd concentration decrease in this time interval (from 0 h to 12 h) for the structures MIP1-Gd and MIP2-Gd is 10.00 mg/L. The subsequent decrease in the concentration occurs more slightly for the both imprinted structures up to 48 h. The mean difference of the Sm concentration decrease in this time interval (from 12 h to 48 h) for the structures MIP1-Sm and MIP2-Sm is 3.42 mg/L.

The values of the Sm and Gd concentrations as they decrease during the metals’ sorption by the imprinted structures are presented in Table 1.

**Figure 5:**
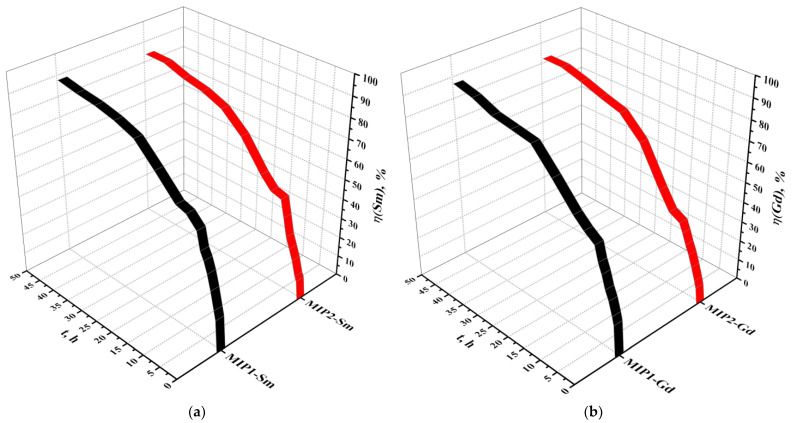
Extraction degrees of samarium (**a**) and gadolinium (**b**) during sorption via molecularly imprinted polymers.

The extraction degrees of samarium and gadolinium by the imprinted structures MIP1-Sm, MIP2-Sm, MIP1-Gd and MIP2-Gd are presented in Figure 5a and Figure 5b, respectively. The extraction degree of samarium increases with time, and a strong increase is observed during the first 24 h of interaction for both imprinted structures. At this time of interaction, the sorption degree is 80.99 for MIP1-Sm and 76.13 for MIP2-Sm, with 90.00% and 88.50% of the total samarium amount sorbed, respectively. The further increase (in the interval of time 24–48 h) in the sorption degree is very slight: for MIP1-Sm, it is 84.63%–86.92%–88.17%–89.99%; for MIP2-Sm, it is 80.17%–82.31%–85.72%–86.02%; the time of interaction is 30 h–36 h–42 h–48 h in both cases. In other words, the increase of extraction degree during the subsequent 24 h is over 5–6%. The sorption degree of gadolinium also increases with time, and a strong increase can be observed in the first 24 h of interaction of the imprinted structures with the salt solution. At 24 h the extraction degree is 79.95% for MIP1-Gd and 75.18% for MIP2-Gd, with 88.93% and 89.72% of the total amount of gadolinium sorbed, respectively. There is subsequently a slight growth of the extraction degree up to the 48 h mark for MIP1-Gd and MIP2-Gd. The increase occurs as follows: for MIP1-Gd, it is 81.56%–83.11%–86.99%–89.11%; for MIP2-Gd, it is 78.13%–81.54%–84.35%–84.54%; the interaction time is 30 h–36 h–42 h–48 h in both cases. The parameter increases over 6–7% during the second day. The phenomenon of a slight increase of the sorption degree during the second day points to equilibrium being achieved between MIP structures and the salt solution.

The values of extraction degrees of samarium and gadolinium are presented in Table 2.

**Figure 6:**
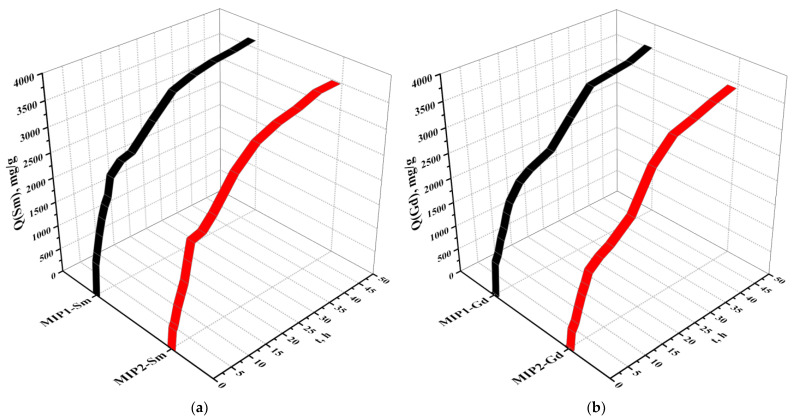
Exchange capacities (in relation to samarium (**a**) and gadolinium (**b**)) of imprinted structures MIP1, MIP2.

Figure 6 shows exchange capacity values (in relation to Sm and Gd) of MIP1-Sm and MIP2-Sm (a) and MIP1-Gd and MIP2-Gd (b). In both cases (sorption of Sm and Gd), a significant increase of the exchange capacity (over 90% of the total values) is observed at 24 h of interaction. The further increase (up to 48 h) of this sorption parameter is insignificant.

The exchange capacity values (in relation to samarium and gadolinium) of the imprinted structures MIP1 and MIP2 are presented in Table 3.

The obtained data show that the non-imprinted structures non-MIP1 and non-MIP2 sorb neither samarium nor gadolinium, which evidences that the synthetized structures MIP1-Sm, MIP2-Sm, MIP1-Gd and MIP2-Gd have selectivity for samarium and gadolinium. The differences in the sorption properties of the structures MIP1 and MIP2 are based on the application of different cross-linking agents during their synthesis. It is supposed that in the case of MIP2, the cross-linking is tighter compared to MIP1, and the sorption process is rather complicated.

### 3.2. Impact of Amount of Template Removal Cycles on Regeneration of MIP Sorption Efficiency

The sorption efficiency studies are based on the amount of template removal cycles. Herein and after, sorption properties (extraction degree, exchange capacity) at 40 template removal cycles (as described in the synthesis procedure) are taken as 100%.

The values of the sorption properties of the imprinted structures MIP1 and MIP2, dependent on the amount of template removal cycles, are presented in Table 4, Table 5, Table 6 and Table 7.

**Figure 7:**
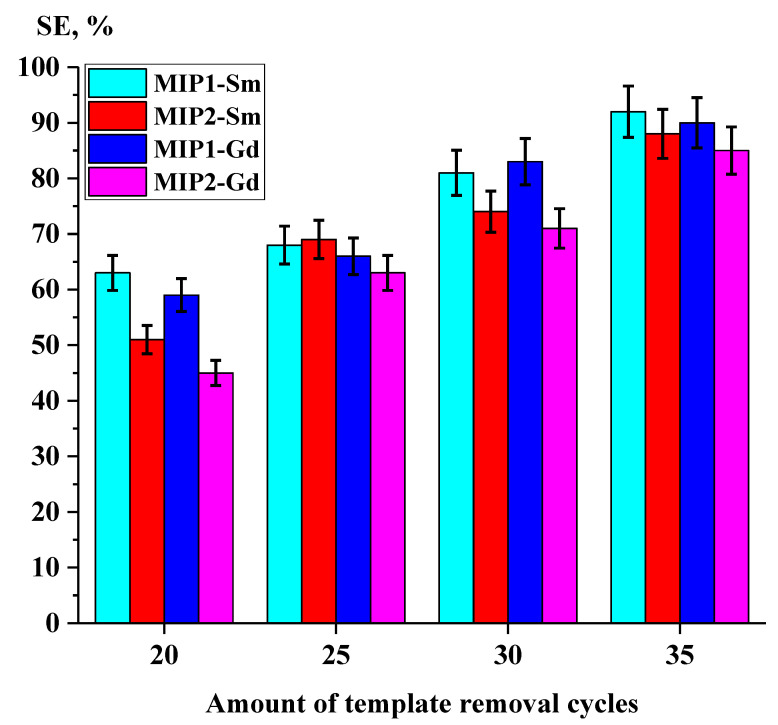
Sorption efficiency of imprinted structures MIP1 and MIP2 depending on amount of template removal cycles.

A comparative analysis of the sorption efficiency of the MIP1 and MIP2 structures, dependent on template removal cycles, is presented in Figure 7. The obtained data show the following sorption efficiency values: for MIP1-Sm, 63% (20 cycles), 68% (25 cycles), 81% (30 cycles) and 92% (35 cycles); for MIP2-Sm, 51% (20 cycles), 69% (25 cycles), 74% (30 cycles) and 88% (35 cycles); for MIP1-Gd, 63% (20 cycles), 59% (25 cycles), 83% (30 cycles) and 90% (35 cycles); and for MIP2-Gd, 45% (20 cycles), 63% (25 cycles), 71% (30 cycles) and 85% (35 cycles). Complete removal of the sorbed REM from the imprinted structure’s matrix is a complicated process. Based on the obtained data, it can be concluded that an increase of the template removal cycles by 5 each time provides an average growth of the sorption efficiency of 11.37%.

### 3.3. Laboratory Experiments on Selective Sorption and Sorption of Sm and Gd

**Figure 8:**
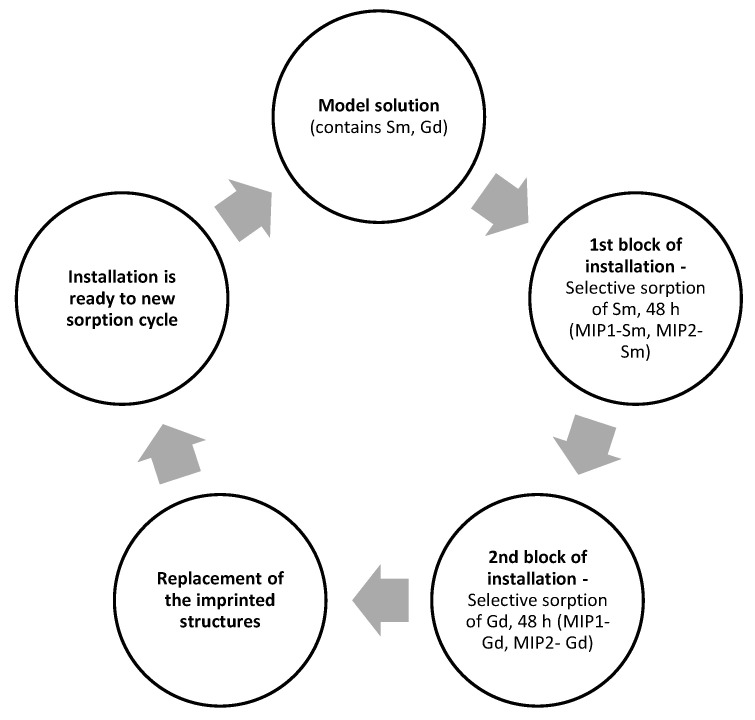
Sorption efficiency of imprinted structures MIP1 and MIP2 in dependence from amount of template removal cycles.

Both imprinted structures (MIP1 and MIP2) are used for the laboratory tests devoted to selective sorption of Sm and Gd. The schematical sorption process during the laboratory tests is shown in Figure 8. As mentioned earlier, laboratory studies on selective sorption of Sm and Gd were carried out with the application of the developed installation (based on two blocks). Each block contains cartridges for the placement of macromolecular dispersions of the imprinted sorbents. The first block is filled with a dispersion of MIP-Sm, and the second one is filled with MIP-Gd. The model solution is pumped into the first block for 48 h (selective sorption of Sm). Subsequently, the solution is pumped into the second block for 48 h (selective sorption of Gd). After the sorption process of both REMs ends, the cartridges with the imprinted structures can be removed and exchanged with other cartridges with MIP structures, and the installation is ready for a new sorption/separation cycle.

The sorption properties of the MIP1 and MIP2 structures during selective sorption of samarium and gadolinium are presented in Table 8, Table 9, Table 10 and Table 11.

As seen from the obtained results of the laboratory tests, the molecular imprinting technique seems to be a promising method for the creation of principally new macromolecular sorbents for the selective sorption and separation of rare-earth metals. The developed MIP1 and MIP2 structures showed effectiveness in selective sorption of the target rare-earth metal, wherein the accompanying sorption of another rare-earth metal was absent. Sorption of the target metal occurs due to the presence of complementary cavities in the polymer matrix of the imprinted structures. As was shown earlier, MIP1 structures are more effective for the sorption of REMs due to the fact that their sorption properties are higher (in comparison with MIP2).

## 4. Conclusions

The developed molecularly imprinted polymers based on MAA and 4VP functional monomers showed good results in the selective sorption/separation of samarium and gadolinium from the common solution. The main advantage of the developed MIPs is the absence of the sorption of accompanying metals (with very close chemical properties). Based on these sorbents, modern effective sorption technologies can be created. As drawbacks, only the complicated procedure of complete desorption of the target metal can be named, along with the relatively high cost of the reactants for MIP synthesis. Despite the mentioned drawbacks, the imprinted sorbents are relevant to be used firstly for modification of existing sorption technologies instead of ion exchangers. Comparison of the synthetized MIPs with the other methods of REM concentration such as extraction of the target REM by phosphoric acid, phosphinic acid and organophosphorus acids [67,68] showed that extraction is high (70–80%), but the drawback that a concentrate of the REM is extracted, and further separation is another complicated step which will make the sorption/separation technology in industry more expensive. The obtained data showed that sorption degree of Sm and Gd by the developed MIPs is almost 90%, and the fact that simultaneous sorption of other metals is absent provides the possibility of using these macromolecular structures as highly effective alternatives to the existing sorption technologies.

## Figures and Tables

**Table 1:** Decreasing values of samarium and gadolinium concentrations via imprinted structures MIP1, MIP2.

t, h	C (Sm) mg/L				C (Gd) mg/L			
	MIP1-Sm	MIP2-Sm	Non-MIP1	Non-MIP2	MIP1-Gd	MIP2-Gd	Non-MIP1	Non-MIP2
0	100.00	100.00	100.00	100.00	100.00	100.00	100.00	100.00
0.5	83.96	90.50	100.00	100.00	84.13	91.99	100.00	100.00
1	79.53	88.35	100.00	100.00	80.09	88.58	100.00	100.00
2	70.75	81.01	100.00	100.00	73.58	82.54	100.00	100.00
3	63.44	75.85	100.00	100.00	69.08	77.02	100.00	100.00
4	57.98	70.70	100.00	100.00	62.72	72.78	100.00	100.00
5	54.33	61.82	100.00	100.00	57.98	67.74	100.00	100.00
6	45.69	53.38	100.00	100.00	50.63	63.53	100.00	100.00
9	40.55	51.91	100.00	100.00	46.95	60.78	100.00	100.00
12	38.99	46.45	100.00	100.00	42.33	52.57	100.00	100.00
18	28.66	32.68	100.00	100.00	30.85	34.41	100.00	100.00
24	19.01	23.87	100.00	100.00	20.05	24.82	100.00	100.00
30	15.37	19.83	100.00	100.00	18.44	21.87	100.00	100.00
36	13.08	17.69	100.00	100.00	16.89	18.46	100.00	100.00
42	11.83	14.28	100.00	100.00	13.01	15.65	100.00	100.00
48	10.01	13.98	100.00	100.00	10.89	15.46	100.00	100.00

**Table 2:** Values of extraction degrees of samarium and gadolinium.

t, h	η (Sm), %				η (Gd), %			
	MIP1-Sm	MIP2-Sm	Non-MIP1	Non-MIP2	MIP1-Gd	MIP2-Gd	Non-MIP1	Non-MIP2
0	0	0	0	0	0	0	0	0
0.5	16.04	9.50	0	0	15.87	8.01	0	0
1	20.47	11.65	0	0	19.91	11.42	0	0
2	29.25	18.99	0	0	26.42	17.46	0	0
3	36.56	24.15	0	0	30.92	22.98	0	0
4	42.02	29.30	0	0	37.28	27.22	0	0
5	45.67	38.18	0	0	42.02	32.26	0	0
6	54.31	46.62	0	0	49.37	36.47	0	0
9	59.45	48.09	0	0	53.05	39.22	0	0
12	61.01	53.55	0	0	57.67	47.43	0	0
18	71.34	67.32	0	0	69.15	65.59	0	0
24	80.99	76.13	0	0	79.95	75.18	0	0
30	84.63	80.17	0	0	81.56	78.13	0	0
36	86.92	82.31	0	0	83.11	81.54	0	0
42	88.17	85.72	0	0	86.99	84.35	0	0
48	89.99	86.02	0	0	89.11	84.54	0	0

**Table 3:** Values of exchange capacity (in relation to samarium and gadolinium) of imprinted structures MIP1 and MIP2.

t, h	Q (Sm), mg/g				Q (Gd), mg/g			
	MIP1-Sm	MIP2-Sm	Non-MIP1	Non-MIP2	MIP1-Gd	MIP2-Gd	Non-MIP1	Non-MIP2
0	0	0	0	0	0	0	0	0
0.5	668.33	395.80	0	0	661.25	333.83	0	0
1	852.92	485.50	0	0	829.58	475.97	0	0
2	1218.75	791.43	0	0	1100.83	727.30	0	0
3	1523.33	1006.37	0	0	1288.33	957.40	0	0
4	1750.83	1220.87	0	0	1553.33	1134.20	0	0
5	1902.92	1590.93	0	0	1750.83	1344.37	0	0
6	2262.92	1942.37	0	0	2057.08	1519.43	0	0
9	2477.08	2003.90	0	0	2210.42	1634.27	0	0
12	2542.08	2231.40	0	0	2402.92	1976.17	0	0
18	2972.50	2805.13	0	0	2881.25	2732.77	0	0
24	3374.58	3172.17	0	0	3331.25	3132.30	0	0
30	3526.25	3340.30	0	0	3398.33	3255.37	0	0
36	3621.67	3429.57	0	0	3462.92	3397.50	0	0
42	3673.75	3571.70	0	0	3624.58	3514.50	0	0
48	3749.58	3584.27	0	0	3712.92	3522.30	0	0

**Table 4:** Values of extraction degrees of samarium after certain amount of template removal cycles.

t, h/ Number of Cycles	η (Sm), %							
	MIP1-Sm				MIP2-Sm			
	20	25	30	35	20	25	30	35
0	0	0	0	0	0	0	0	0
0.5	10.11	10.91	12.99	14.76	4.84	6.55	7.03	8.36
1	12.90	13.92	16.58	18.83	5.94	8.04	8.62	10.25
2	18.43	19.89	23.69	26.91	9.69	13.11	14.06	16.72
3	23.03	24.86	29.61	33.64	12.32	16.67	17.87	21.25
4	26.47	28.57	34.04	38.66	14.94	20.22	21.68	25.78
5	28.77	31.06	36.99	42.02	19.47	26.35	28.25	33.60
6	34.22	36.93	43.99	49.97	23.77	32.17	34.50	41.02
9	37.45	40.43	48.15	54.69	24.53	33.18	35.59	42.32
12	38.44	41.49	49.42	56.13	27.31	36.95	39.63	47.13
18	44.94	48.51	57.79	65.63	34.33	46.45	49.82	59.24
24	51.02	55.07	65.60	74.51	38.83	52.53	56.34	67.00
30	53.32	57.55	68.55	77.86	40.89	55.32	59.32	70.55
36	54.76	59.11	70.41	79.97	41.98	56.79	60.91	72.43
42	55.55	59.96	71.42	81.12	43.72	59.15	63.43	75.43
48	56.69	61.19	72.89	82.79	43.87	59.36	63.66	75.70

**Table 5:** Values of exchange capacity (in relation to samarium) after certain amount of template removal cycles.

t, h/ Number of Cycles	Q (Sm), mg/g							
	MIP1-Sm				MIP2-Sm			
	20	25	30	35	20	25	30	35
0	0	0	0	0	0	0	0	0
0.5	421.05	454.47	541.35	614.87	201.86	273.10	292.89	348.30
1	537.34	579.98	690.86	784.68	247.61	335.00	359.27	427.24
2	767.81	828.75	987.19	1121.25	403.63	546.09	585.66	696.46
3	959.70	1035.87	1233.90	1401.47	513.25	694.39	744.71	885.60
4	1103.03	1190.57	1418.18	1610.77	622.64	842.40	903.44	1074.36
5	1198.84	1293.98	1541.36	1750.68	811.38	1097.74	1177.29	1400.02
6	1425.64	1538.78	1832.96	2081.88	990.61	1340.23	1437.35	1709.28
9	1560.56	1684.42	2006.44	2278.92	1021.99	1382.69	1482.89	1763.43
12	1601.51	1728.62	2059.09	2338.72	1138.01	1539.67	1651.24	1963.63
18	1872.68	2021.30	2407.73	2734.70	1430.62	1935.54	2075.80	2468.52
24	2125.99	2294.72	2733.41	3104.62	1617.81	2188.80	2347.40	2791.51
30	2221.54	2397.85	2856.26	3244.15	1703.55	2304.81	2471.82	2939.46
36	2281.65	2462.73	2933.55	3331.93	1749.08	2366.40	2537.88	3018.02
42	2314.46	2498.15	2975.74	3379.85	1821.57	2464.47	2643.06	3143.10
48	2362.24	2549.72	3037.16	3449.62	1827.98	2473.14	2652.36	3154.15

**Table 6:** Values of extraction degree of gadolinium after certain amount of template removal cycles.

t, h/ Number of Cycles	η (Gd), %							
	MIP1-Gd				MIP2-Gd			
	20	25	30	35	20	25	30	35
0	0	0	0	0	0	0	0	0
0.5	9.36	10.47	13.17	14.28	3.61	5.05	5.69	6.81
1	11.75	13.14	16.53	17.92	5.14	7.20	8.11	9.71
2	15.59	17.44	21.93	23.78	7.85	11.00	12.39	14.84
3	18.24	20.41	25.66	27.83	10.34	14.48	16.31	19.53
4	22.00	24.60	30.94	33.55	12.25	17.15	19.33	23.14
5	24.79	27.73	34.88	37.82	14.52	20.33	22.91	27.43
6	29.13	32.58	40.98	44.43	16.41	22.97	25.89	31.00
9	31.30	35.01	44.03	47.75	17.65	24.71	27.85	33.34
12	34.03	38.06	47.87	51.90	21.34	29.88	33.67	40.31
18	40.80	45.64	57.39	62.24	29.51	41.32	46.57	55.75
24	47.17	52.77	66.36	71.96	33.83	47.36	53.37	63.90
30	48.12	53.83	67.69	73.40	35.16	49.22	55.47	66.41
36	49.03	54.85	68.98	74.80	36.69	51.37	57.89	69.31
42	51.32	57.41	72.20	78.29	37.96	53.14	59.89	71.70
48	52.57	58.81	73.96	80.20	38.04	53.26	60.02	71.85

**Table 7:** Values of exchange capacity (in relation to gadolinium) after certain amount of template removal cycles.

t, h/ Number of Cycles	Q (Gd), mg/g							
	MIP1-Gd				MIP2-Gd			
	20	25	30	i35	20	25	30	35
0	0	0	0	0	0	0	0	0
0.5	390.14	436.43	548.84	595.13	150.23	210.32	237.02	283.76
1	489.45	547.53	688.55	746.63	214.19	299.86	337.94	404.57
2	649.49	726.55	913.69	990.75	327.29	458.20	516.38	618.21
3	760.12	850.30	1069.32	1159.50	430.83	603.16	679.75	813.79
4	916.47	1025.20	1289.27	1398.00	510.39	714.55	805.28	964.07
5	1032.99	1155.55	1453.19	1575.75	604.97	846.95	954.50	1142.71
6	1213.68	1357.68	1707.38	1851.38	683.75	957.24	1078.80	1291.52
9	1304.15	1458.88	1834.65	1989.38	735.42	1029.59	1160.33	1389.13
12	1417.72	1585.93	1994.42	2162.63	889.28	1244.99	1403.08	1679.74
18	1699.94	1901.63	2391.44	2593.13	1229.75	1721.64	1940.26	2322.85
24	1965.44	2198.63	2764.94	2998.13	1409.54	1973.35	2223.93	2662.46
30	2005.02	2242.90	2820.62	3058.50	1464.92	2050.88	2311.31	2767.06
36	2043.12	2285.53	2874.22	3116.63	1528.88	2140.43	2412.23	2887.88
42	2138.50	2392.23	3008.40	3262.13	1581.53	2214.14	2495.30	2987.33
48	2190.62	2450.53	3081.72	3341.63	1585.04	2219.05	2500.83	2993.96

**Table 8:** Sorption properties of the MIP1-Sm and MIP1-Gd structures during selective sorption of samarium.

t, h	Selective Sorption of Sm			
	MIP1-Sm		MIP1-Gd	
	η (Sm), %	Q (Sm), mg/g	η (Sm), %	Q (Sm), mg/g
0	0	0	0	0
0.5	16.04	668.33	0	0
1	20.47	852.92	0	0
2	29.25	1218.75	0	0
3	36.56	1523.33	0	0
4	42.02	1750.83	0	0
5	45.67	1902.92	0	0
6	54.31	2262.92	0	0
9	59.45	2477.08	0	0
12	61.01	2542.08	0	0
18	71.34	2972.50	0	0
24	80.99	3374.58	0	0
30	84.63	3526.25	0	0
36	86.92	3621.67	0	0
42	88.17	3673.75	0	0
48	89.99	3749.58	0	0

**Table 9:** Sorption properties of the MIP1-Sm and MIP1-Gd structures during selective sorption of gadolinium.

t, h	Selective Sorption of Gd			
	MIP1-Sm		MIP1-Gd	
	η (Sm), %	Q (Sm), mg/g	η (Sm), %	Q (Sm), mg/g
0	0	0	0	0
0.5	0	0	15.87	661.25
1	0	0	19.91	829.58
2	0	0	26.42	1100.83
3	0	0	30.92	1288.33
4	0	0	37.28	1553.33
5	0	0	42.02	1750.83
6	0	0	49.37	2057.08
9	0	0	53.05	2210.42
12	0	0	57.67	2402.92
18	0	0	69.15	2881.25
24	0	0	79.95	3331.25
30	0	0	81.56	3398.33
36	0	0	83.11	3462.92
42	0	0	86.99	3624.58
48	0	0	89.11	3712.92

**Table 10:** Sorption properties of the MIP2-Sm and MIP2-Gd structures during selective sorption of samarium.

t, h	Selective Sorption of Sm			
	MIP2-Sm		MIP2-Gd	
	η (Sm), %	Q (Sm), mg/g	η (Sm), %	Q (Sm), mg/g
0	0	0	0	0
0.5	9.50	395.80	0	0
1	11.65	485.50	0	0
2	18.99	791.43	0	0
3	24.15	1006.37	0	0
4	29.30	1220.87	0	0
5	38.18	1590.93	0	0
6	46.62	1942.37	0	0
9	48.09	2003.90	0	0
12	53.55	2231.40	0	0
18	67.32	2805.13	0	0
24	76.13	3172.17	0	0
30	80.17	3340.30	0	0
36	82.31	3429.57	0	0
42	85.72	3571.70	0	0
48	86.02	3584.27	0	0

**Table 11:** Sorption properties of the MIP2-Sm and MIP2-Gd structures during selective sorption of gadolinium.

t, h	Selective Sorption of Gd			
	MIP2-Sm		MIP2-Gd	
	η (Sm), %	Q (Sm), mg/g	η (Sm), %	Q (Sm), mg/g
0	0	0	0	0
0.5	0	0	8.01	333.83
1	0	0	11.42	475.97
2	0	0	17.46	727.30
3	0	0	22.98	957.40
4	0	0	27.22	1134.20
5	0	0	32.26	1344.37
6	0	0	36.47	1519.43
9	0	0	39.22	1634.27
12	0	0	47.43	1976.17
18	0	0	65.59	2732.77
24	0	0	75.18	3132.30
30	0	0	78.13	3255.37
36	0	0	81.54	3397.50
42	0	0	84.35	3514.50
48	0	0	84.54	3522.30

## Data Availability

The data presented in this study are available upon request from the corresponding author.
